# A Model Milk Supply

**Published:** 1904-12-10

**Authors:** G. F. McCleary

**Affiliations:** Medical Officer of Health, Battersea


### Hospital Clinics.

### A MODEL MILK SUPPLY.

Abstract of a Paper read before the Wandsworth Division of the British Medical Association, by G. F. McCleary, M.D.(Cantab), D.P.H., Medical Officer of Health, Battersea.

With the increasing appreciation of the importance and value of milk as a food has come increased knowledge of the dangers that exist in our present methods of milk production and distribution, and the reform of the milk supply may be regarded as the most pressing of the problems that preventive medicine is called upon to solve. It is useful, therefore, to note what progress has been made in other countries and especially in Copenhagen where, according to the testimony of many observers, milk is supplied under better conditions than those which obtain in any other European city.

The milk supply of Copenhagen is largely in the hands of three companies: the Kjobenhaous Maelkeforsyning (Copenhagen Milk Supply Company), the Danish Milk Company, and the Trifolium Milk Supply Company, and in the operations of these companies the interest of the city's milk supply lies. The first is the oldest and much the best of the three, and as its methods have been copied to some extent by its rivals some account of these methods will indicate the lines on which the reform of Copenhagen's milk supply has proceeded.

The Kjobenhaous Maelkeforsyning was established some 25 years ago by Mr. G. Busch, who from the first has been the honorary manager of the company. Mr. Busch is a man of exceptional ability and force of character, and the success of the company is almost entirely due to his energy, initiative, and public spirit. Before the company was established the milk supply of the city was in the hands of spirit distillers whose practice it was to keep a herd of cows at the distillery, feed the animals on the distillery refuse, and combine the sale of milk and spirits on the same premises. There are still some 18 of these distillery cowsheds in Copenhagen. I visited seven during my stay in Denmark last summer, and found them to be as insanitary as any cowshed I have seen in this country. When the distillers controlled the milk trade, it sometimes happened that milk was supplied only on the condition that spirits also were purchased on the premises, and Mr. Busch's attention was first turned to the milk question on hearing a workman complain that he had been refused milk at a certain distillery because he did not buy his spirits there. Mr. Busch determined to start a milk supply that should provide pure milk at the lowest possible price and that the matter of profit-making should be a secondary consideration. He was supported by some public-spirited men in Copenhagen and started his company. The profits are limited to 5 per cent., anything over this must be devoted to extending the business.

The aim of the company is to supply at a low price "pure milk from healthy cows." Sterilisation is not employed, nor is pasteurisation, except for a small supply of specially prepared infants' milk. The company relies upon the observance of strict cleanliness accompanied by a liberal use of ice. It has no farms of its own, but contracts with about thirty farmers for the supply of milk. The conditions of contract are severe, but in return for their observance the farmer is paid a better price for his milk than he can get elsewhere. The main provisions of the contract are as follows:—

The milk must be drawn from healthy cows only. If a cow is suffering from tuberculosis it must be separated at once from the rest of the herd, and got rid of as soon as possible. The feeding of the cows is prescribed in detail. Stall-feeding in summer is absolutely prohibited; the cows must be fed in the open air upon clover or grass, and if it is necessary to give dry food this must be eaten in the field. In the winter, when the cows are in the sheds, the food is chiefly hay and bran, but oilcake, carrots, and sugar beets are permitted in small specified quantities, and a small amount of sunflower cake, which is now a popular cattle food in Denmark, is also allowed. Brewers' grains and distillery refuse, turnips, etc., are absolutely forbidden. Each farm is visited at least once a fortnight by one of the company's veterinary surgeons who examines every cow on the farm. In order to encourage the early detection of udder disease a reward is offered to any milker who reports a lesion of the udder.

The farmer must watch over the health of all who reside or work on his farm, and should any case of infectious disease occur he must at once report the fact to the Company and withhold his milk, which will, however, be paid for as usual if the conditions are fully complied with. Conditions as to cleanliness are laid down. In autumn, before the cows are taken into the sheds, their udders, tails, and hind quarters are clipped. The milker has to wash his hands after milking each cow, and the milk must be carefully strained.

Great importance is attached to rapid cooling of the milk. The farmer must be provided with a Lawrence cooler, which he can hire from the Company, and must stock a large quantity of ice. The milk must be cooled down to 40° F., or lower, as soon as possible after it is drawn from the cow.

The milk is conveyed to the city in sealed churns provided by the company, and most of it travels in the company's own railway vans, which are fitted with ice-tanks.

Although the company thus endeavours to secure the best possible conditions at the source of the supply, Mr. Busch regards the control of the farms as the weak point in his scheme, and he thinks a really satisfactory system of control cannot be secured without direct ownership of the farms. When, however, the milk arrives at the company's premises in the city it comes under Mr. Busch's own direct control, and from that time the methods are, on the whole, excellent, as I can testify from personal observation. The premises cover over two acres of ground. The working-rooms are very large, exceedingly well-lighted and ventilated, and are cool even in the hottest weather. The most scrupulous cleanliness prevails all over the premises. A striking feature is the huge ice-house, which holds over two million pounds of ice. This quantity is laid in every winter, the ice being obtained from a lake which formerly served as one of the city reservoirs. The milk arrives at a special railway platform on the company's premises at 10 P.M. Each churn is weighed, and, after unsealing, two samples of milk are taken out, one for tasting, the other for analysis; the temperature of the milk is taken, and on all my visits it was below 50° F. The milk is then set down in ice, and later, filtered by upward filtration through three layers of gravel and six layers of fine cloth. After passing through the filter the children's milk is bottled, and the bottles set down in ice until they are distributed to the consumer. The milk for ordinary consumption is received into churns which are sealed and placed in the distributing vans, the doors of which are locked by an officer of the company. The milk is drawn from the churn through a tap which projects from the lower part of the churn through an aperture in the side of the van. By these precautions adulteration on the milk-round becomes a matter of great difficulty. The price of the milk is 16 öre the litre (about $2\frac{3}{4}$d. a quart). The greatest care is observed in cleaning the bottles and churns. Two women are employed solely in examining the cleansed bottles to see that no particle of milk or dirt remains. The churns are cleansed as follows: The churn is first rinsed with cold water and drained, then hot soda solution is poured in, and the interior well scrubbed with a stiff brush. Mr. Busch thinks the length of the English churn a grave defect, as it prevents a person with an arm of normal length from reaching the bottom with a brush. The Danish churn holds only 50 litres, and every part can be easily reached. The soda solution is then poured out and the churn placed on a large wheel, which revolves in a tank containing a saturated solution of lime. After the outside has been well scrubbed the churn is inverted over a jet of boiling water and then allowed to drain.

The two other large companies in Copenhagen, the Danish Milk Company and the Trifolium Company, are purely commercial undertakings. All the milk supplied by the former is pasteurised; the retail supply is bottled, but a large amount is sent out in bulk to hotels, restaurants, etc. The Trifolium Company's supply is all sent out in bottles, but no preserving process other than cold storage is employed. Both these companies obtain their milk from farmers, and profess to secure the observance of conditions as strict as those imposed by the Kjobenhaous Maelkeforsyning. This may be so, but certainly the methods employed in the treatment of the milk in the city will not bear comparison with those of the latter company. The interest of the Copenhagen milk supply lies chiefly in the operations of the Kjobenhaous Maelkeforsyning, and the success of that organisation is due to the fact that it is directed by a man of exceptional powers who has chosen to devote his life to the problem of bringing good milk within the reach of the poor.

